# The Immune Deficiency and Dysregulation Activity (IDDA2.1 ‘*Kaleidoscope’*) Score and Other Clinical Measures in Inborn Errors of Immunity

**DOI:** 10.1007/s10875-021-01177-2

**Published:** 2021-11-19

**Authors:** Markus G. Seidel, Victoria K. Tesch, Linlin Yang, Fabian Hauck, Anna Lena Horn, Maria Anna Smolle, Franz Quehenberger, Martin Benesch

**Affiliations:** 1 Division of Pediatric Hematology-Oncology, Department of Pediatrics and Adolescent Medicine, Medical University of Graz, Auenbruggerplatz 38, A-8036 Graz, Austria; 2 Research Unit for Pediatric Hematology and Immunology, Medical University of Graz, Graz, Austria; 3 Department of Clinical Immunology, Royal Free London NHS Foundation Trust, London, NW3 2PF UK; 4 Institute for Immunity and Transplantation, University College London, London, NW3 2PF UK; 5 Division of Pediatric Immunology and Rheumatology, Department of Pediatrics, Dr. Von Hauner Children’s Hospital, University Hospital, Ludwig-Maximilians-Universität München, Munich, Germany; 6 Department of Orthopedics and Trauma, Medical University of Graz, Graz, Austria; 7 Institute for Medical Informatics, Statistics, and Documentation, Medical University Graz, Graz, Austria

**Keywords:** Inborn error of immunity (IEI), primary immunodeficiency (PID), primary immune regulatory disorder (PIRD), score, scale, measure, index, algorithm, heatmap, spider chart

## Abstract

Quantifying the phenotypic features of rare diseases such as inborn errors of immunity (IEI) helps clinicians make diagnoses, classify disorders, and objectify the disease severity at its first presentation as well as during therapy and follow-up. Furthermore, it may allow cross-sectional and cohort comparisons and support treatment decisions such as an evaluation for transplantation. On the basis of a literature review, we provide a descriptive comparison of ten selected scores and measures frequently used in IEI and divide these into three categories: (1) *diagnostic tools* (for Hyper-IgE syndrome, hemophagocytic lymphohistiocytosis, and Wiskott-Aldrich syndrome), (2) *morbidity and disease activity measures* (for common variable immune deficiency [CVID], profound combined immune deficiency, CTLA-4 haploinsufficiency, immune deficiency and dysregulation activity [IDDA], IPEX organ impairment, and the autoinflammatory disease activity index), and (3) *treatment stratification* scores (shown for hypogammaglobulinemia). The depth of preclinical and statistical validations varies among the presented tools, and disease-inherent and user-dependent factors complicate their broader application. To support a comparable, standardized evaluation for prospective monitoring of diseases with immune dysregulation, we propose the IDDA2.1 score (comprising 22 parameters on a 2–5-step scale) as a simple yet comprehensive and powerful tool. Originally developed for use in a retrospective study in LRBA deficiency, this new version may be applied to all IEI with immune dysregulation. Reviewing published aggregate cohort data from hundreds of patients, the IDDA *kaleidoscope function* is presented for 18 exemplary IEI as an instructive phenotype–pattern visualization tool, and an unsupervised, hierarchically clustered heatmap mathematically confirms similarities and differences in their phenotype expression profiles.

**Supplementary Information:**

The online version contains supplementary material available at 10.1007/s10875-021-01177-2.

## Introduction

In clinical oncology and rheumatology, staging, grading, and diagnostic criteria are routinely applied to define the diagnosis or a subtype of a disorder and consequently to determine the appropriate management of a patient and the treatment of a disease. Similarly, scores that include physiological, biometrical, laboratory and medical history data are applied to assess the probability of survival of patients in intensive care units (broadly or more or less organ-specific) or to quantify end-stage organ damage, e.g., liver disease. However, in the immunodeficiency clinic, such algorithms and guidelines are less frequently available. Most patients with IEI are managed on a descriptive–diagnosis and problem-directed — more or less individual — basis, with the exception of patients with severe combined immunodeficiency (SCID) and certain other distinct inborn errors of immunity (IEI), where a clear genotype–phenotype correlation and the therapeutic consequences of a (mostly genetic) diagnosis (e.g*.*, a targeted therapy, gene therapy, or hematopoietic stem cell transplantation [HSCT]) have been established. This management is especially challenging in immunodeficient patients with accompanying syndromic or complex features, such as immune dysregulation with autoimmunity and inflammation, a predisposition for malignancy, bleeding, or hemophagocytosis, or other increased risks. To categorize IEI phenotypes into most likely (sub-)diagnoses, document the natural clinical course of the disease, evaluate treatment responses, and scientifically improve management recommendations for these individuals, various attempts have been made to quantify and standardize the assessment of a disease phenotype and category by using scores, indexes, scales, and other measures.

In the first part, we provide an overview of various types of clinical scores currently used in IEI. Without claiming to be exhaustive, we briefly describe ten representative tools and compare their scopes, applications, and limitations. The second part presents an updated version of the immune deficiency and dysregulation activity (IDDA) score. Originally developed to perform a retrospective comparison of the clinical courses and disease burden of patients with a deficiency of the lipopolysaccharide and beige-like anchor protein (LRBA)[1] to compare long-term outcomes with or without HSCT, the presented version IDDA2.1 is designed to be a useful measure for prospectively assessing the immune deficiency and dysregulation activity of all patients with IEI and immune dysregulation, including primary immune regulatory disorders (PIRDs) as well as many combined immunodeficiencies and predominantly antibody deficiencies (categories I-IV of the IEI classification of the International Union of Immunological Societies [IUIS] [2, 3]). We demonstrate the power of its parameters by using unsupervised hierarchical clustering across various IEI with immune dysregulation and discuss advantages of the IDDA2.1 score, particular differences as compared to other measures in IEI, and the future potential as a predictive tool for disease (sub-)categorization, allowing for the deduction of specific screening recommendations on the basis of those complications expected for the respective IEI, as well as serving as a guide to support phenotype-targeted diagnostic and treatment decisions in patients without a genetic diagnosis and monitoring their effects. Finally, the *kaleidoscope function* of the IDDA2.1 score is applied to 18 IEI with immune dysregulation of various IUIS categories, demonstrating its educational value.

## Routine Applications and Brief Description of Representative Types of Clinical Scores in IEI

**Fig. 1:**
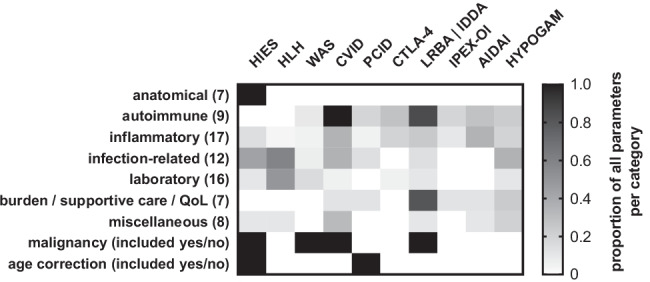
Schematic comparison of scores and measures routinely used in the IEI clinic. Please refer to Table 1 and the original publications for more details. The selection of scores represents an exemplary choice of the authors

On the basis of a literature review performed in January 2021, we selected ten representative instruments that are used as measures, scores, scales, or indexes (hereafter generally referred to as “scores”) to assess clinical features and the disease activity of IEI quantitatively and qualitatively. Among these frequently used tools, we defined three categories of clinical scores for IEI with different fields of applications and consequently varying sets of parameters (Table 1, Fig. 1). These include the following:

1. *Diagnostic scores* comprising a set of disease-specific weighted criteria can increase the likelihood of making a specific diagnosis or sub-classifying a diagnosis. Three examples of tools assisting diagnosis-making are the Hyper-IgE syndrome (HIES) score, which was originally designed for autosomal dominant STAT3-LOF syndrome [4, 5], and the ‘diagnostic guidelines’ and the ‘H score’ used for primary and secondary hemophagocytic lymphohistiocytosis (HLH), respectively [6–8] (Table 1). These are widely accepted and statistically validated tools that are used to corroborate making a diagnosis of a suspected IEI or defining the likelihood of the presence of the respective syndrome. Of note, these measures are not designed to guide a differential diagnostic work-up in presence of only one or few of their predominant symptoms. Their examples, however, illustrate the challenges associated with defining an entity by applying clinical diagnostic criteria independently of the existence/awareness of a genetic background. Namely, the definition of the HIES of genetic origins other than mutations in *STAT3* or differences between the familial or acquired forms of hemophagocytic syndromes required modifications of the original definitions. For instance, in the case of deficiency of DOCK8, the HIES score was adapted to reflect additional and unique features of this other type of HIES [9]. While the HIES score is predominantly based on preexisting or aggravating anatomical features in an individual, their infectious history and laboratory values, and includes a correction for a young age, the hemophagocytosis scores rely mainly on laboratory values, fever, and organomegaly (Table 1, Fig. 1). The diagnostic tool for familial hemophagocytic lymphohistiocytosis was defined by the Histiocyte Society [8], and differs slightly from other sets of diagnostic criteria that have been proposed for secondary HLH [6, 10, 11], as well as from the H score for acquired HLH, which is also applied to correct for potentially causative or modifying factors, such as underlying malignancy, infection, or existing pharmacological immunosuppression [7].
  An instrument used to classify a subcategory of a diagnosis by taking into account the presence of certain features or complications can help to define the subtype of an IEI. The Wiskott-Aldrich syndrome (WAS) score is a typical example of the instruments that are used to categorize a diagnosis into subtypes [12–14]. In this X-linked syndrome, typically characterized by the triad of infection, microthrombocytopenia, and eczema, the phenotypic spectrum covers a wide range that usually does not change over time within one patient. This spectrum includes “classic/severe” WAS ± autoimmunity categories, various categories in X-linked thrombocytopenia (intermittent or persistent, ± eczema and immunodeficiency) and in X-linked neutropenia (mildest form) on a scale ranging from 0 to 5. Although classifying patients from mild to severe forms of WAS and used in clinical cohort studies, this score has been shown to be of relatively little predictive relevance with regard to the development of autoimmunity or malignancy [15].
2. *Morbidity and disease activity measures* can be applied to grade the disease severity and burden, which may change during the clinical course of one patient. These scores are typically re-assessed at every clinical visit or scheduled time points of a patient with a defined IEI. Different results for these scores may reflect a separate subtype of the disorder or, more often, a distinct phase of the disease the patient is going through. One example is the collection of common variable immune deficiency (CVID) or CVID complication scores [16–18]; these scores can be used to quantify the severity of organ involvement and the accumulating inflammatory or autoimmune and even treatment-related complications in a patient. Points are added for every feature, and the scores are observed over the patient history. For profound combined immunodeficiency (P-CID), a morbidity measure was introduced that is part of an ongoing prospective study, which includes bacterial, viral, and opportunistic infections, immune-mediated cytopenia or other signs of immune dysregulation, and lung involvement, and corrects for the time of exposure (number of events divided by patient’s age); additional information on the patient-reported quality of life is analyzed separately [19]. A more disease-specific score than those used for CVID or P-CID is the CTLA-4 haploinsufficiency with autoimmune infiltration (CHAI) morbidity measure, which is being used in the ongoing abatacept-CHAI study (*B. Grimbacher, personal communication*). The CHAI morbidity measure quantifies the organ involvement in detail by translating specific lab values including results of FACS analyses, imaging studies, or physiological functional results into points. The item list includes many non-routine investigations and thus requires a structured patient visit with all tests to be planned beforehand. A related disease with many features similar to CTLA-4 haploinsufficiency is LRBA deficiency, for which we recently developed a simple but comprehensive score that includes both a quantification of the severity of infectious complications and of features of immune dysregulation, and the need for supportive care, performance scale, hospitalization days, IgG substitution therapy, and nutritional interventions to reflect the entire disease burden [1]. This score with its differences from other presented measures, named *immune deficiency and dysregulation activity (IDDA) score*, is explained in more detail below. Another, even much simpler but effective, disease-specific score is the assessment of organ involvement in IPEX syndrome (immune dysregulation, polyendocrinopathy, enteropathy, X-linked) on a scale from 0 to 5 (1 point per prognosis-relevant organ involvement) [20]. This score was used as part of a highly thorough and comprehensive retrospective analysis of patient- and treatment-related factors in IPEX syndrome and was identified as being significantly linked to the HSCT outcome of these patients [20].
  Assessing the disease severity and burden is also an important factor in the management of patients with autoinflammatory syndromes (AIS) such as hereditary fever syndromes. The *autoinflammatory disease activity index* (AIDAI) represents a different type of tool based on a patient-maintained disease diary that was developed and statistically validated in a multistep process; this index has been designed to suit many of the known AIS [21, 22], a fact that is corroborated by its application, even if in slightly adapted forms, in current clinical trials [23, 24].
3. Finally, *treatment stratification scoring systems* may be used to support decisions on therapy. In IEI, a widely relevant example is the score to guide the initiation of immunoglobulin therapy based on laboratory parameters and the clinical history of adult patients with hypogammaglobulinemia (including CVID; Table 1 and Fig. 1) [25]. With a similar aim, a more complex version of a treatment algorithm for patients with primary antibody deficiencies was published as interdisciplinary consensus- and evidence-based guideline by the *Association of Scientific Medical Societies in Germany (AWMF)* and updated recently [26, 27]. Although the latter instrument includes many similar criteria extracted from the history, clinical status, and laboratory parameters of hypogammaglobulinemic patients like the above-mentioned score, it was not designed as a ‘score’ but as a step-by-step algorithm that includes degrees of evidence for each criterion.

**Table 1:** Parameter overview of 10 selected clinical scores and measures used in IEI

Parameters	HIES^1^	HLH^2^	WAS^3^	CVID^4^	P-CID^5^	CTLA-4^6^	LRBA-IDDA^7^	IPEX-OI^8^	AIDAI^9^	Hypo–gam^10^
Anatomical^11^										
Skoliosis	X									
Facial dysmorphia	X									
Inadequate fractures	X									
Mid line defect, high palate	X									
Nose width	X									
Hyperextensible joints	X									
Retained primary teeth	X									
Autoimmunity										
n.f.s.^12^			X	X	X					X
Endocrinopathies				X			X			
CNS incl. Neurological n.f.s.^13^				X		X	X			
Eye^13^				X			X		X	
Skin^13^				X		X	X		X	
*Lung (see below)*	*-*	*-*	*-*	*-*	*-*	*-*	*-*	*-*	*-*	*-*
*Gut (see below)*	*-*	*-*	*-*	*-*	*-*	*-*	*-*	*-*	*-*	*-*
Liver				X			X	X		
Kidney				X			X	X		
Immune cytopenias				X	X	X	X			X
Musculoskeletal^13^				X			X		X	
Inflammatory^1^										
Fever		X							X	
Splenomegaly^13^		X		X		X	X			X
Lymphadenopathy, non-malignant lymphoproliferative disease				X		X	X			X
Parenchymal lung disease, GLILD, LIP	X			X	X	X	X			
Granuloma (except GLILD)				X			X			
Carditis				X						
Vasculitis				X						
Enteropathy, celiac disease, gastritis, inflammatory bowel disease, protein-losing enteropathy				X		X	X	X		X
Neonatal exanthema	X									
Eczema n.f.s	X		X							
Respiratory impairment n.f.s								X		
Abdominal pain									X	
Nausea, Vomiting									X	
Diarrhoea									X	X
Headaches									X	
Chest pain									X	
Painful nodes									X	
Infection-related										
Abscesses	X (skin, organ)									
Pneumonia	X									X
Bronchiectasis				X						X
ENT and respiratory infections	X			X						X
Candidiasis	X									
Other invasive infections	X			X						X
Infections n.f.s			X		X		X			
Infections viral or opportunistic n.f.s					X					
Gut incl. Helicobacter pylori				X						X
CNS				X						
Fatal infection	X									
Asymptomatic/chronic infestation							X			
Laboratory										
Genetic diagnosis		X								
IgE	X									
Eosinophil count	X									
Cytopenias (usually not autoimmune)		X	X							
Small platelets			X							
Hypertrigliceridemia		X								
Hypofibrinogenemia		X								
Hyperferritinemia		X								
Increased sIL2R		X				X				
Hemophagocytosis		X		X			X			
Myelodysplasia			X							
Reduced NK cell activity		X								
Serum aspartate aminotransferase (AST)		X								
Cellular markers of immune activation, naivety, memory formation						X				
Hypogammaglobulinemia (see below IVIG or SCIG therapy)							X			X
Specific antibody concentrations										X
QoL, supportive measures, performance scales										
Patient-reported quality of life, symptom burden					X				X	
Failure to thrive, malnutrition, weight loss				X		X	X	X		X
Karnofsky/Lansky scale							X			
Hospitalization (except ICU)							X			X
ICU, mechanical ventilation							X			
IVIG or SCIG therapy							X			
Nutrition/dietary status							X			
Other										
Allergies				X						
Vascular disease n.f.s	X									
Known underlying immunosuppression		in H score								
Iatrogenic complications				X						
Amyloidosis				X						
Other organ dysfunction n.f.s							X			X
Antibiotic (courses/year)										X
Pain relief drugs taken									X	
Malignancy										
Malignancy n.f.s		in FHL: absence	X	X			X			
Lymphoma	X									
Age correction	X				X					

^*1*^*,* extracted from the HIES score for STAT3LOF [4, 5]

^*2*^*,* a sum of parameters for familial hemophagocytic lymphohistiocytosis (FHL) and secondary hemophagocytic syndrome (H score) [7, 8]

^*3*^, summarized from [13]

^*4*^, extracted from the modified version by Ameratunga, 2018 [18]

^*5*^, taken from [19]

^*6*^, extracted from the ABACHAI trial that evaluates the safety and efficacy of abatacept in patients with CTLA- 4 insufficiency and LRBA deficiency (000,972–40 EU Clinical Trial Registry)

^*7*^, modified from [1]

^*8*^, IPEX organ involvement score, taken from [20]

^*9*^, summarized from [21, 22]

^*10*^*,* Hypogam, slightly simplified from *hypogammaglobulinemia treatment (immunoglobulin therapy) indication scoring system *[25]

^*11*^*,* in the wide sense, including also results of functional defects that cause secondary changes

^*12*^*,* n.f.s., not further specified

^*13*^*,* overlap between infectious, autoimmune and inflammatory pathogenesis of features n.f.s

*ENT* ear nose throat

*CNS* central nervous system

*QoL* quality of life

ICU intensive care unit

*IVIG, SCIG* intravenous or subcutaneous immunoglobulin therapy

The proportional comparison of items and parameter categories used in each of these ten scores are depicted in Fig. 1 and listed in Table 1 (the original publication of each of the selected representative scores is cited for reference and to provide access to more detailed information). While type 1 scores from the list above rely on yes/no criteria or a semiquantitative “staging” to support making a diagnosis, most of the type 2 and 3 scores are supposed to be applied repeatedly to grade the actual disease activity status of a patient by adding scores for the affected organs, symptoms, complications, or other features. Some of these scores can even quantify the severity of each criterion. The latter quality of a score is needed if it should serve as a monitoring tool to document the clinical course of a patient in longitudinal studies, e.g., in prospective drug trials or for registry purposes. Naturally, the extent and application of a score in IEI reflects the difficulties associated with making either a diagnosis or managing affected patients with a certain disorder. Thus, using the established scores in the routine management of patients with IEI has a practical and beneficial awareness-raising effect, i.e., to prevent mistakes and negligence when applying the recommended monitoring measures, as the respective scores are often based on the experience of large patient series that encompass the full phenotypic spectrum of a rare disease known at that time.

## The Immune Deficiency and Dysregulation Activity (IDDA2.1 ‘*Kaleidoscope’*) Score

The IDDA2.1 score is designed as a simple, physician-reported outcome measure tool and is used to assess the severity of the involvement of 12 organ systems in immune dysregulation (e.g., autoimmunity, autoinflammation, lymphoproliferation, granuloma formation) and two other clinical features (failure to thrive and severe infections) that are scored on a 5-step scale from 0 to 4 (indicating 0 = absent; 1 = mild, transient, not requiring treatment; 2 = moderate, intermittent therapy needed; 3 = severe, continuous therapy needed; 4 = life-threatening, refractory, irreversible; Table 2). In addition, the proportion of hospitalization days (differentially weighted whether at a regular ward or an intensive care unit) and the need for supportive therapies and care are noted (nutrition status and route, IgG substitution therapy, Table 2). Furthermore, “any other organ or immune dysfunction” and “any relevant chronic or recurring infestation or infection” are added to the score; the sum is then divided by the performance scale (Karnovsky or Lansky [28, 29]; see Table 2 for detailed calculation). Occurrence of a malignancy is noted separately but is not quantified within the numeric score. Parameters of the original version of the IDDA score were chosen to reflect the entire clinical spectrum of LRBA deficiency according to previously published case series and reviews. In the newly adapted version 2.1, only two major changes were introduced: the item ‘hemophagocytosis’ was added as additional possible feature of immune dysregulation to extend the utility to practically all PIRDs or IEI with immune dysregulation, and the calculation method (formula) was slightly modified to correct for very low performance scales (Table 2, bottom line). In contrast to the presented other 9 measures in IEI, many of which focus on disease-specific anatomical (HIES), infectious (CVID, and many others) or inflammatory (HLH, CVID, AIDAI) parameters, and, especially, to the other *morbidity and disease activity measures*, the IDDA2.1 score specifically addresses all most relevant features of immune dysregulation in IEI in a semiquantified, comprehensive, organ-specific manner, and includes indicators of the actual disease and treatment burden, while maintaining relative simplicity. In fact, its composition is designed to allow documentation by retrospective chart review by a medical documentarist after a patient visit, as performed even as part of a registry study, without the need to obtain specific laboratory values or questionnaires, or to schedule specific imaging or physiological tests ahead. Assessing patient-reported symptom scores or quality of life/outcome measures by questionnaires (as in the AIDAI or P-CID studies, respectively) was omitted for the same reason as was the inclusion of results from particular functional, laboratory, or imaging analyses (as, e.g., in the CHAI score) — practicability. Nevertheless, recording the presence, severity, or absence of each parameter and the IDDA score sum over time and different treatment phases of a patient should enable to draw a wide-ranging, highly defined picture of the clinical status and course of a patient with a PIRD or IEI with immune dysregulation. Although relatively simple by design (grading from 0 to 4, see above), limitations of the scoring were encountered, for example, quantifying the severity of infections. A molluscum or mucocutaneous HSV infection of a DOCK8-deficient patient will be assessed differently than in a patient with CVID, and a severe pneumococcal pneumonia of a hypogammaglobulinemic patient will be treated differently from an EBV-linked systemic lymphoproliferative and inflammatory response syndrome in CD27 deficiency, although all of them will be scored as 3° or 4° infection (range 0–4°) depending on treatment response, reversibility, and whether the event was life-threatening. Furthermore, difficulties may arise in the judgement, whether organ lesions are caused by an infection or by infection-triggered immune dysregulation and as such should be counted as organ involvement (e.g., lung or CNS granulomata) — the distinction of which may be easy in certain and difficult or impossible in other occasions. However, the general focus of graded organ involvement caused by immune dysregulation of the IDDA score sum and its longitudinal course are not affected by these differences.

**Table 2:** The immune deficiency and dysregulation activity (IDDA2.1) score

	**Parameters (for grading 0–4°, see below**^**1**^**; line titles serve as examples)**
1	Autoimmune (AI) cytopenia
2	Hemophagocytosis | HLH (according to clinical AND lab criteria of the HS)
3	Enteropathy | IBD
4	Lymphoproliferation | splenomegaly | hepatomegaly
5	Parenchymal lung disease | LIP | GLILD
6	Skin or eye manifestations | eczema, uveitis, alopecia, vitiligo, other
7	Granulomatous disease in any organ (except GLILD)
8	Endocrinopathy | IDDM, thyreoiditis, other
9	Arthritis | other musculoskeletal
10	AI-hepatitis | cholangitis | pancreatitis
11	Glomerulonephritis | nephropathy, tubulopathy
12	Neurologic manifestations of immune dysregulation | CNS autoimmunity, inflammation, vasculitis
13	Failure to thrive | malresorption, wasting
14	Severe infections | opportunistic (excl. chronic infestation, see below)
	**Other factors and symptoms (will multiply or add to the IDDAscore)**^**2**^
15	Karnovsky / Lansky Performance Scale (%)
16	Hospitalization (days out of 100 days; including day clinic stays, excl. intensive care unit [ICU])
17	Mechanical ventilation or other ICU measures (days out of 100 days; except elective procedures)
18	Immunoglobulin substitution therapy | hypogammaglobulinemia
19	Any relevant chronic or recurring infestation/infection (*e.g.*, Norovirus, EBV…)
20	Any other organ dysfunction / malady (*e.g.*, cardiomyopathy, kidney failure, allergies…)
21	Nutrition / dietary status and habits
**22**	**Malignancy (separately noted, not added to numeric score)**
**Formula for IDDA2.1 score total (Excel® format)** = (SUM(line1:line14) + IF(line16 < 40;line16*0.1;4) + IF(line17 < 10;line17*0.8;8) + SUM(line18:line21))*IF(line15 > 29;150/line15;6)	

^1^, grading for lines 1–14: 0, absent; 1, mild, transient, not requiring treatment; 2, moderate, intermittent therapy needed; 3, severe, continuous therapy needed; 4, life-threatening, refractory, irreversible

^2^, lines 15–17 are percentages, lines 18–21 are scored as follows: line 18 (0, no; 2, sporadic; 3[iv], regularly IVIG; 3[sc], regularly SCIG); line 19 (0, no; 1, asymptomatic infestation; 2, oligosymptomatic recurring infection; 3, recurring symptomatic infection requiring on/off treatment; 4, chronic infection requiring permanent treatment or refractory infection, only score worst if more than one microbial agents are relevant); line 20 (e.g*.*, hepatopathy, cardiomyopathy, kidney failure; please quantify if possible: 0, no organopathy; 1, mild transient dysfunction; 2, chronic mild dysfunction; 3, moderate-severe dysfunction; 4, clinically compromising dysfunction requiring treatment or replacement therapy, only score worst if more than one organ is involved); line 21 (0, normal; 1, modified disease-adjusted; 2, part-formula medically advised; 3, tube-feeding and/or full-formula or partial parenteral nutrition (irregularly); 4, total parenteral nutrition)

The IDDA2.1 score may be used for a wide range of applications in clinical documentation. Supplementary Fig. 1 shows examples of the first version of the IDDA score as it was used in a retrospective study of patients with LRBA deficiency that compared the clinical courses before, during, and after different treatments over time or in patients with residual LRBA protein expression as compared to those with absent LRBA protein [1], heatmaps illustrate the severity of each organ involvement before and after a certain therapeutic measure was applied, spaghetti plots may be used for longitudinal comparisons (Supplementary Fig. 1), and Kaplan–Meier curves may be plotted to compare outcomes of cohorts with different IDDA starting scores (not shown) [1].

**Fig. 2:**
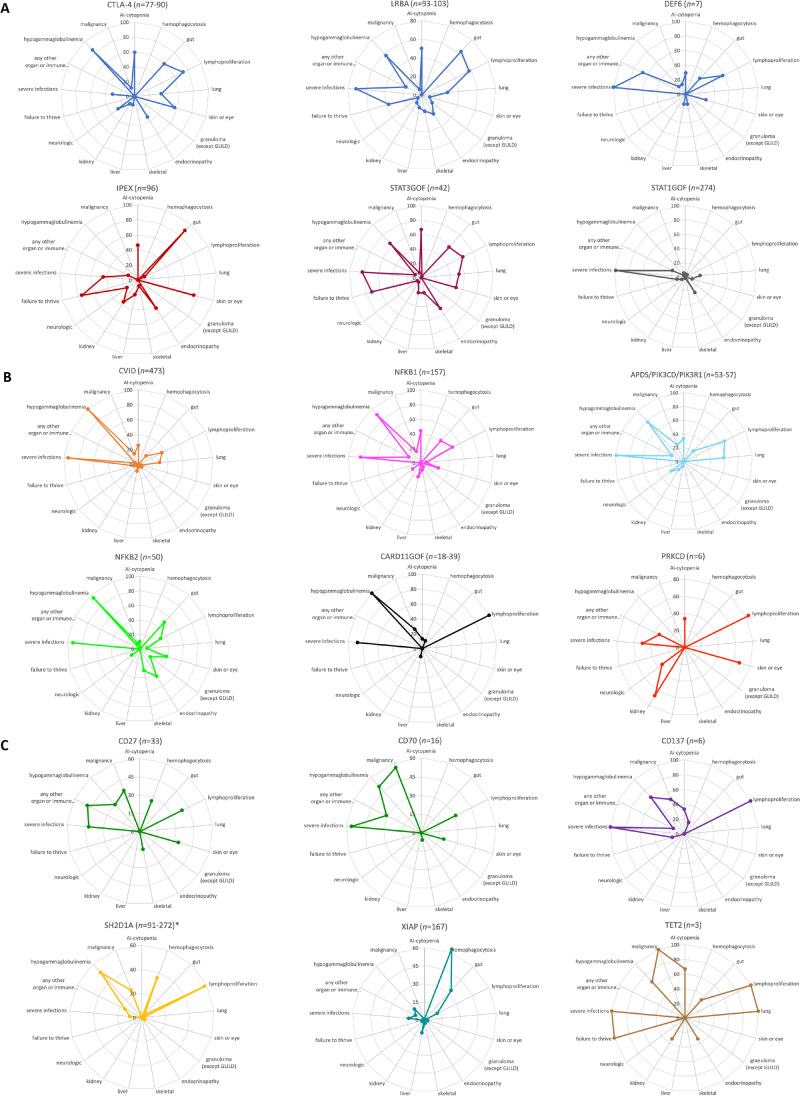
Immune deficiency and dysregulation phenotype patterns visualized by the IDDA2.1 *kaleidoscope* score for 18 exemplary IEI. The *kaleidoscope* function makes use of 17 out of 22 parameters documented in the IDDA score (terms #1–14; #20; #18; and #22, see Table 2), reduced to qualitative information about organ involvement and other features in a patient or a patient cohort, plotted according to the fixed order of parameters in a radar (spider) chart on 17 *y*-axes arranged in a circle. **A** Primary immune regulatory disorders, modified from [30] and supplemented with additional IEI, with data derived from reviews, case series, or large cohorts [1, 20, 31–36]; **B** Predominantly antibody deficiencies and combined immunodeficiencies [37–43]; **C** Diseases of immune regulation with EBV-susceptibility [44–50]. Data for part of the CARD11GOF and all of the XIAP plots were derived from unpublished data to appear in Hauck et al., 2021, and a manuscript in preparation by Yang and Burns et al., 2021, respectively. The patient numbers presented in the title of each plot may vary slightly regarding some features that were not available from all patients, but are always presented as a percentage on 17 *y*-axes. In the regular (22-parametric) IDDA score originally developed for LRBA deficiency [1], each criterion is semi-quantified per patient from 0 to 4◦, please refer to Table 2 for details. The full-length *y*-axis titles are autoimmune (AI)-cytopenia; hemophagocytosis | HLH (according to clinical AND lab criteria of the Histiocyte Society); gut, enteropathy | IBD (inflammatory bowel disease); lymphoproliferation | splenomegaly | hepatomegaly; lung, parenchymal lung disease | LIP (lymphocytic interstitial pneumonitis)| GLILD (granulomatous lymphocytic interstitial lung disease); skin or eye manifestations | eczema, uveitis, alopecia, vitiligo, other; granulomatous disease in any organ (other than GLILD); endocrinopathy | IDDM (insulin-dependent diabetes mellitus), thyroiditis, other; skeletal, arthritis | other musculoskeletal manifestations; liver, AI-hepatitis | cholangitis | pancreatitis; kidney, glomerulonephritis | nephropathy, tubulopathy; neurologic manifestations; failure to thrive | malresorption, wasting; severe infections | severe or opportunistic infections (excl. asymptomatic chronic infestation; excluding “EBV-susceptibility”); any other organ or immune dysfunction/malady (e.g., cardiomyopathy, kidney failure, autoinflammation, allergy); hypogammaglobulinemia and/or immunoglobulin substitution therapy; malignancy, lymphoma (separately added to IDDA score, not included in the score calculation); *, the footnote (asterisk) in SH2DA1 deficiency (XLP1) should indicate that, although this topic is debatable, the liver and kidney involvement in fulminant infectious mononucleosis was not counted under immune dysregulation (#10–11), likewise the CNS involvement in patients with HLH and XLP1 was not counted as organ-specific immune dysregulation (#12), and aplastic anemia observed in patients with XLP1 was not counted as autoimmune cytopenia (#1) to distinguish their pathogenesis from “primarily” immune-mediated organ manifestations in PIRDs

**Fig. 3:**
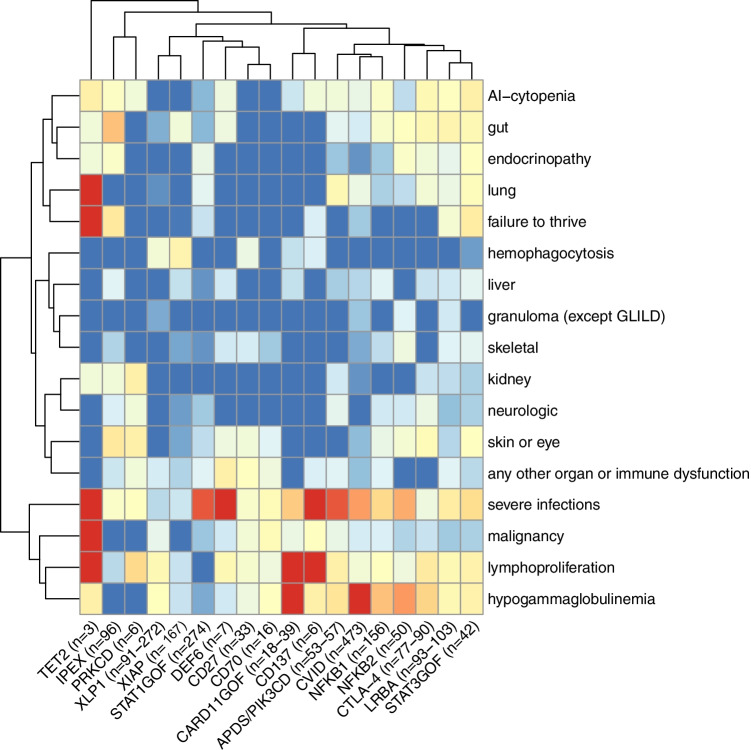
‘Phenotype expression array’ showing results of unsupervised hierarchical clustering of 18 IEI with immune dysregulation according to the IDDA2.1 parameter list. The same patient cohorts were analyzed as shown and referenced in Fig. 2. The clustered heatmap was created by using the R package pheatmap 1.0.12 (Raivo Kolde, 2019). The hierarchical clustering was the result of complete linkage based on Euclidean distances (after logistic transformation truncated at 1%). Red boxes indicate high frequency, blue boxes low frequency. “Pedigrees” indicate the calculated similarities or distances; not taking into account the relative frequency of patients evaluated in the original data collection (see numbers next to the IEI diagnosis). Therefore, due to very low patient numbers (< 10), phenotype data for DEF6, PRKCD, CD137, and TET2 deficiencies are less reliable than those for other IEI with higher patient numbers

A new accompanying feature that can be used to document the phenotype of patients or patient cohorts by using the IDDA2.1 score is the *kaleidoscope function*. It relies on the same data collected for the IDDA2.1 score, but instead of calculating a numerical score, the frequency of the presence of 17 of the 22 features/items within a patient cohort is plotted in a circular arrangement (spider or radar chart, listing the parameters in a fixed order as in Table 2), resulting in a recognizable, relatively pathway-specific pattern. To illustrate this arrangement, we applied the IDDA kaleidoscope score to 18 different IEI, some of which shared the same impaired pathway (e.g*.*, CTLA-4 haploinsufficiency, LRBA, and DEF6 deficiencies, see Fig. 2A; data are derived from published patient series or reviews that are cited in the legend). Others were chosen due to their frequency or their novelty. For educational purposes, we grouped the 18 IEI into predominantly regulatory T cell disorders (Tregopathies), CVID)/CVID-like disorders, and PIRDs with EBV susceptibility (Fig. 3A, B, and C, respectively). Of note, the kaleidoscope patterns of CTLA-4 haploinsufficiency, LRBA, and DEF6 deficiencies look very similar, but differ from those of IPEX syndrome or CVID (Fig. 3A, B). On the other hand, the patterns for CVID, NFKB1, and NFKB2 deficiency resemble each other closely (Fig. 3B), possibly reflecting a large proportion of undetected NFKB1 and NFKB2 deficiencies among CVID cohorts, as do the patterns for the receptor-ligand pair CD27 and CD70 deficiencies (Fig. 3C). Expectedly, the ‘kaleidoscope pattern’ of XLP1 differs from that of XLP2/XIAP deficiency, whereas, independent of the direct pathomechanism, that of STAT3GOF looks very much alike that of LRBA deficiency. Thus, an immediate conclusion of a similarity of the biomechanistic basis and thus treatment recommendation cannot be drawn from this first, retrospectively generated plot. An alternative way to present these data could be a heatmap (Supplementary Fig. 2), which may be preferred for pattern recognition by people used to interpret array data. To verify the grouping of IEI based on clinical experience and to prioritize the clinical features according to their relevance in this set of 18 IEI, we performed an unsupervised hierarchical clustering analysis of the same data which yielded clusters of diseases according to their feature frequency profiles (neighbors in the horizontal order and clustering, Fig. 3) that is strikingly similar but not identical to that of our clinical and “visual” order applied in the spider charts shown in Fig. 2. The heatmap derived from this clustering analysis reveals “pedigrees” of the most relevant and discriminative clinical parameters among the IDDA2.1 list for this set of 18 IEI (vertical order/clustering, Fig. 3). Because we used aggregated cohort data from large series or reviews to generate Fig. 2 and lack most of the raw (single patient) data, we were unable to calculate similarities of the kaleidoscope patterns statistically, *e.g.*, in principal component analyses. Although individual patients will, naturally, be unlikely to resemble complete congruence in these phenotype expression analyses from aggregate data, the IDDA2.1 *kaleidoscope function* may gain importance if applied regularly and fed into a machine learning algorithm as discussed below. For educational purposes, the present catalogue of spider charts or heatmaps may be extended to any IEI with immune dysregulation.

## Conclusions and Future Perspectives

A standardized, quantitative definition of the clinical phenotype of an IEI in its entirety and its consequences on the patient’s quality of life, need for supportive care, and disease burden is relevant both in disease- or drug-specific studies as well as in prospective patient registry studies to learn about the natural course of the disease.

Ensuring the comparability and transferability of scoring results between specific disorders and between patients with undiagnosed IEI, either cross-sectionally or over time, is an advantage of more general disease scores as opposed to single-disease-focused scores. However, in order to propose a clinical score that covers more than one distinct disease and likewise measures disease activity, several obstacles must first be overcome. First, the balance has to be maintained between simplicity and feasibility, on the one hand, and detail and specificity on the other hand, while still avoiding the need to refer to a meticulous handbook or user manual. It is difficult to define which organ damage should be included into the immune dysregulation-dependent scoring process, and which should be included only into “other organ dysfunction,” e.g., the differentiation between whether parenchymal changes in an organ should be attributed to recurring invasive infections or represent the result of inflammation and dysregulated organ infiltration. The same vagueness applies to the different reasons of lymphoproliferation whether “genuine” (e.g., in autoimmune lymphoproliferative syndrome) or as result of chronic lymphadenopathy, furthermore, to hepatosplenomegaly in the context of HLH or CVID, or to cytopenia associated with splenomegaly, etc. (see also the asterisk-marked footnote at XLP1 in Fig. 3C). Similarly, any definitions may not always be uniformly understandable or may show varying levels of resolution, e.g., the terms “autoimmunity” or “cytopenia” may not be very helpful, given the fact that either the target organ or the pathomechanism remained undefined, thus impairing the correct scoring. Furthermore, user-dependent factors, such as variations in the assessment of disease severity, may compromise the comparability of data. Second, the phenotype of a specific IEI with a typically increased vulnerability in a defined organ (e.g., colitis), towards certain infectious microorganisms (e.g., herpes viruses, mycobacteria, candidiasis), or with another characteristic clinical feature (e.g., allergy, vasculitis) may not be depicted proportionally through the use of a more general score such as the IDDA score. In these cases, a disease-specific score may yield more granular results and thus be more adequately applied or it should be used in addition to a standard score. Third, the disease activity depends on the treatment status of a patient. Therefore, documenting a score of the above-mentioned category 2 (severity/disease activity), such as the IDDA score, should automatically involve its timely correlation with the current therapy of a patient and ideally be repeated at regular intervals.

One main goal of the human phenotype ontology (HPO) project is to standardize the terminology used to describe phenotypic characteristics, the definitions of disease features and manifestations, symptoms, laboratory abnormalities, and other clinical aspects. This project has recently been extended to include many IEI [51–55]. An initial attempt to include HPO terms into the IDDA score was temporarily aborted, because we aimed to achieve the utmost simplicity when incorporating the 22 parameters into the ESID registry database. Nevertheless, the inclusion of HPO codes into the background of data entry fields, possibly with refined sub-items, might increase the accuracy and resolution of clinical data documentation. An international initiative is ongoing to define these terms in more detail and to extend the applicability of HPO terms in IEI [54].

In the AIDAI, for example, significant efforts have been made to achieve statistical validation [21], and this validation might be achieved with respect to other scores as well. However, the composition of a score in IEI will mostly be based on clinical observations of the two- or three-digit patient numbers only, and its application will be used for descriptive purposes rather than for strictly exclusive decision-making or stratifications. Because a clinical score in IEI will never be a stand-alone criterion for making or excluding a diagnosis or a treatment decision, and because largely overlapping phenotypes are observed in many of these extremely rare diseases, it will be difficult to provide statistical validation for many of the purposes these scores may fulfill. With regard to the IDDA2.1 score, an open science prospective study proposal has been approved and launched in the context of the ESID registry.

We propose that, collecting IDDA2.1 score data and applying the IDDA kaleidoscope function in a standardized manner in patients with PIRDs in the future may allow validation of pattern comparisons, algorithm-assisted recognition, and potentially even phenotype-based diagnosis prediction of PIRDs. To this end, the IDDA2.1 score has been implemented in the European Society for Immunodeficiencies (ESID) registry database [56] as an optional module to serve as a basis for a prospective study using monogenic IEIs as a learning cohorts. With enough data from known IEIs and, ideally, multiple patient time points under different therapies, this should allow to generate a machine-learning-powered tool which should eventually assist clinicians in making diagnoses, monitor expectable risk organs, and support phenotype-driven, "semi-targeted" treatment decisions for immune dysregulation in undiagnosed patients. In this study, attempts will be made to rank the relevance of items collected in the ESID registry according to their predictive power regarding the diagnosis, progression, and treatment response, e.g., by applying logistic regression models, time-dependent Cox-regression models, and the nomograms based on these, as described elsewhere [57, 58]. This should enable the ESID registry’s composition and application to be further strengthened and refined. When taking this approach, we will apply unsupervised machine learning algorithms to detect similarities in patterns in training cohorts consisting of patients with known monogenic IEI.

If scores in IEI are regarded as assistive measures and their application is restricted to specific studies or in specific institutions consistently, they may be very helpful in clinical and clinical research practice. Using and applying a “score standard” in the clinical practice in IEI, as shown with the IDDA2.1 score for PIRDs and other IEI with immune dysregulation, may prove useful as an educational tool and enhance the awareness of the complexity of phenotypes and complications, ultimately improving patient outcomes.

## Supplementary Information

Below is the link to the electronic supplementary material.

Supplementary file1 (DOCX 1106 kb)

## Data Availability

Not applicable.

## Abbreviations

AI-cytopenia: Autoimmune cytopenia
AIDAI: Autoinflammatory disease activity index
AIS: Autoinflammatory syndrome
APDS: Activated phosphoinositide 3-kinase δ syndrome
CARD11-GOF: Caspase recruitment domain-containing protein 11 gain-of function
CHAI: CTLA-4 insufficiency with autoimmune infiltration
CNS: Central nervous system
CTLA-4: Cytotoxic T-lymphocyte antigen-4
CVID: Common variable immunodeficiency
DEF6: Differentially expressed in FDCP 6 homolog
DOCK8: Dedicator of cytokinesis 8
GLILD: Granulomatous lymphocytic interstitial lung disease
HIES: Hyper-IgE syndrome
HLH: Hemophagocytic lymphohistiocytosis
HPO: Human phenotype ontology
IBD: Inflammatory bowel disease
ICU: Intensive care unit
IDDA score: Immune deficiency and dysregulation activity score
IDDM: Insulin-dependent diabetes mellitus
IEI: Inborn error of immunity
IPEX syndrome: Immunodeficiency polyendocrinopathy enteropathy, X-linked syndrome
IUIS: International Union of Immunological Societies
IVIG, SCIG: Intravenous or subcutaneous immunoglobulin G therapy
LIP: Lymphocytic interstitial pneumonitis
LRBA: Lipopolysaccharide-responsive beige-like anchor protein
NFKB1/NFKB2: Nuclear factor kappa B1/2
NK cells: Natural killer cells
P-CID: Profound combined immunodeficiency
PID: Primary immunodeficiency
PIK3CD / PIK3R1: Phosphoinositide 3-kinase genes
PIRD: Primary immune regulatory disorder
PRKCD: Protein kinase C delta
SCID: Severe combined immunodeficiency
STAT3-LOF / STAT1-LOF: Signal transducer and activator of transcription-3 (-1) loss-of-function
TET2: Ten eleven translocation (TET) methylcytosine dioxygenase 2
Treg cells: Regulatory T cells
Tregopathies: Regulatory T cell disorders
WAS: Wiskott-Aldrich syndrome
XIAP, XLP2 (gene: BIRC4): X-linked inhibitor of apoptosis
XLP1 (gene: SH2D1A): X-linked lymphoproliferative syndrome type 1
