# Correction to: The Immune Deficiency and Dysregulation Activity (IDDA2.1 ‘*Kaleidoscope’*) Score and Other Clinical Measures in Inborn Errors of Immunity

**DOI:** 10.1007/s10875-022-01220-w

**Published:** 2022-02-09

**Authors:** Markus G. Seidel, Victoria K. Tesch, Linlin Yang, Fabian Hauck, Anna Lena Horn, Maria Anna Smolle, Franz Quehenberger, Martin Benesch

**Affiliations:** 1 Division of Pediatric Hematology-Oncology, Department of Pediatrics and Adolescent Medicine, Medical University of Graz, Auenbruggerplatz 38, A-8036 Graz, Austria; 2 Research Unit for Pediatric Hematology and Immunology, Medical University of Graz, Graz, Austria; 3 Department of Clinical Immunology, Royal Free London NHS Foundation Trust, London, NW3 2PF UK; 4 Institute for Immunity and Transplantation, University College London, London, NW3 2PF UK; 5 Division of Pediatric Immunology and Rheumatology, Department of Pediatrics, Dr. Von Hauner Children’s Hospital, University Hospital, Ludwig-Maximilians-Universitat Munchen, Munich, Germany; 6 Department of Orthopedics and Trauma, Medical University of Graz, Graz, Austria; 7 Institute for Medical Informatics, Statistics, and Documentation, Medical University Graz, Graz, Austria

**Correction to: Journal of Clinical Immunology**

10.1007/s10875-021-01177-2

Due to typesetting mistake, the published PDF and HTML versions did not match.

The original version has been corrected.

