# Supplementary material for: The Immune Deficiency and Dysregulation Activity (IDDA2.1 ‘Kaleidoscope’) Score and Other Clinical Measures in Inborn Errors of Immunity
Source: J Clin Immunol. 2021 Nov 19;42(3):484–98. doi: 10.1007/s10875-021-01177-2 (PMC9016022; doi:10.1007/s10875-021-01177-2)
Supplement: Supplementary file 1 — Supplementary file1 (DOCX 1106 kb) [file 10875_2021_1177_MOESM1_ESM.docx]

**Supplementary Material**

**The immune deficiency and dysregulation activity (IDDA2.1 ‘*kaleidoscope’*) score and other clinical measures in inborn errors of immunity**

Markus G. Seidel^1, 2, 8^, Victoria K. Tesch^1, 2^, Linlin Yang^3, 4^, Fabian Hauck^5^, Anna Lena Horn^2^, Maria Anna Smolle^6^, Franz Quehenberger^7^, Martin Benesch^1^

Supplementary Figure 1 with Legend.

Supplementary Figure 2 with Legend.

*Supplementary Figure 1.* **Example applications of the IDDA score (modified from *Tesch et al., 2020)*.** Graphical data visualization of various possible applications of the IDDA score, taken and modified from the first version developed for a retrospective study on LRBA deficiency(1). The heatmap shown in the upper left-hand panel shows grading of organ manifestations and other features as documented at every IDDA score evaluation, while the other plots show the score sums per time point per patient for different analyses. Please refer to the original publication for LRBA deficiency-specific details of the data.

*Supplementary Figure 2.* **Heatmap showing percentages of phenotypical features of 18 exemplary IEI with immune dysregulation.** Data are the same as in the main Figure 2, the order of clinical features is fixed according to the IDDA2.1 score (Table 2). Data sources are provided in detail in the legend to Figure 2. IEI with similar patterns of phenotypical features are indicated by brackets marked as “A1” (part of panel A, Figure 2A), “B” for panel B (Figure 2), and part of panel C from Figure 2C (“C1”).
